# In Situ Analysis of Historical Preservation Fluids in Sealed Containers with Spatially Offset Raman Spectroscopy

**DOI:** 10.1021/acsomega.5c09045

**Published:** 2026-01-13

**Authors:** Ana Blanco, Wren Montgomery, Sam Walker, Chelsea McKibbin, Robert Stokes, Pavel Matousek, Sara Mosca

**Affiliations:** † 6426 Agilent Technologies LDA U.K., Becquerel Avenue, Didcot OX11 0RA, U.K.; ‡ Science Innovation Platforms, Department of Science, 4946 Natural History Museum, Cromwell Road, London SW7 5BD, U.K.; § Central Laser Facility, Research Complex at Harwell, STFC Rutherford Appleton Laboratory, UKRI, Harwell Campus, Harwell OX11 0QX, U.K.; ∥ Department of Physics and Astronomy, University of Exeter, Exeter EX4 4QL, U.K.

## Abstract

Understanding and monitoring of preservation fluids is essential for ensuring the long-term integrity of fluid-preserved specimens in natural history collections. However, analytical characterization remains challenging, particularly as noninvasive and portable solutions are required. This study presents the first in situ application of spatially offset Raman spectroscopy (SORS) combined with multivariate analysis for classifying preservation fluids in historical biological specimens. A total of 46 fluid-preserved samples from the Natural History Museum, London, UK were analyzed under collection conditions using a portable device. The method accurately identified the preservation fluids in 78.5% of cases and showed partial agreement in another 15%, often with visually similar or chemically complex solutions. Only 3 samples (6.5%) were misclassified or unclassified. SORS spectra revealed main excipients and low-concentration additives, with classification ambiguities typically arising from fluorescence effects or secondary components not included in the calibration set. Additionally, the proposed approach allowed distinguishing between different types of glass (and/or plastic) container providing potential insights into fluid–container interactions and historical storage conditions. Overall, the approach demonstrated high chemical specificity, sensitivity to mixtures, and broad potential for conservation and collection management.

## Graphical abstract

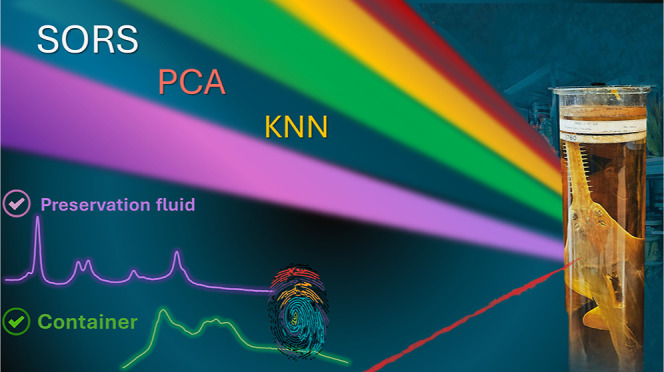

## Introduction

Fluid-preserved biological specimens are significant in heritage sciences, specifically within natural history collections, offering invaluable insights into biodiversity, anatomy, and scientific history. The long-term conservation of these specimens depends critically on the chemical stability of the preservation fluids in which they are stored.^1,2^ The chemical compositions of preservation fluids have historically varied widely due to evolving protocols, recipes, and conservation and curatorial interventions.^3^ Identifying and monitoring preservation fluids are essential for specimen conservation planning.^4^ The most common preservative preparations are alcohol-based,^5^ particularly ethanol and methanol, with an increase in concentration as distillation techniques improved.^6^ The discovery of the fixative properties of formaldehyde in the late 19th century^7^ introduced a new standard, particularly for anatomical and pathological specimens. These main excipients were frequently used in combination with a wide range of additives, including sugar, camphor, sea salt, mercuric chloride, picric acid, and arsenic, to enhance preservation and minimize microbial decay.^3,8^ Many historical formulations reflect this diversity. For instance, Ruysch’s solution included aromatic spices (clove, pepper, cardamom) in an ethanol–water base.^9^ Owen’s fluid consists of water, sea salt, and alum, sometimes with mercuric chloride, and its composition closely resembles Goadby’s solution, used in British collections.^10^ Further examples include Bouin’s solution, which is a mixture of formaldehyde, picric acid, and acetic acid that was developed for optimal tissue fixation, and histological contrast.^11^ Similarly, Kaiserling’s method, originally designed to maintain the natural color of specimens, involved sequential immersion in formaldehyde, potassium nitrate, and glycerine.^12^ Over time, the variability in recipes through both deliberate substitutions and undocumented interventions, has led to considerable heterogeneity across collections, with mixtures of ethanol, methanol, glycerol, and formaldehyde commonly encountered in unknown proportions, further altered by potential evaporation and contamination over time. This complexity underscores the need for noninvasive methods deployable in situ to identify and quantify fluid components without opening containers, which could compromise the specimen and expose operators to toxic substances.^2,13^ Many traditional methods for fluid identification, such as density measurement, colorimetric strip testing, or gas chromatography–mass spectrometry, require direct access to the fluid.^14,15^ This often entails opening sealed containers, risking specimen exposure to air and contamination,^1^ as well as compromising historical information embedded in the original sealing and container materials. Raman spectroscopy, thanks to its high a molecular specificity,^16^ is a valuable technique for the noninvasive identification of chemical compounds and their relative concentrations without requiring direct sampling or preparation.^17^ Cersoy et al.^8^ have demonstrated that conventional Raman microscopy can successfully distinguish between various historical preservation fluid formulations. However, its application was limited by signal interference from the container (e.g., jar fluorescence) and the need to operate within a controlled laboratory setting. As a result, the method was restricted to specimens that could be safely transported and accommodated within the microscope’s sample chamber. To address these limitations, our previous work^18^ introduced a combined approach using spatially offset Raman spectroscopy (SORS)^19^ and principal component analysis (PCA). This was a proof-of-concept laboratory based study performed on artificially prepared solutions contained in a single historic jar with no animal specimens present. Unlike traditional Raman, SORS enhances subsurface signal detection by introducing a spatial separation between the laser illumination and the collection point,^20^ effectively reducing fluorescence and Raman signal interference from the container itself.^18,21,22^

Here we present the first in situ chemical characterization of historical preservation fluids using the SORS method within a museum setting. We used a hand-held SORS device combined with multivariate analysis (i.e., PCA, *k*-nearest neighbors classifier algorithm (KNN-classifier) and multivariate curve resolution (MCR)) to examine and classify 46 fluid-preserved specimens from the Natural History Museum (NHM), London, UK. Despite challenges such as variations in containers, environmental factors, and the complex nature of aged fluids (prone to degradation phenomena, specimen leakage, fat release, discoloration, and residual biological signals from specimen), the method successfully differentiated between different fluid types and relative concentrations. Moreover, the SORS approach allows the isolation of the spectral signal of the container from that of the fluid, enabling the classification of container materials (e.g., soda-lime glass, borosilicate, lead glass, acrylics, and plastics), offering valuable insights into historical storage practices. The developed method supports preventive conservation and collection care without disturbing the specimens or their containers. In summary, the main objectives of this study were to evaluate the feasibility of SORS for noninvasive identification of preservation fluids under realistic collection conditions and assess the potential of the method to inform curatorial documentation and conservation practices.

## Experimental Section

### Liquid Solutions—Calibration

Building on our previous work,^18,23^ we expanded the calibration data set^23^ by preparing a set of solutions that simulated historical preservation fluids. Table 1 lists the 20 solutions (C1–C20), which include varied concentrations of glycerol, ethanol, methanol, and formaldehyde, as well as complex mixtures that mimic well-known historic recipes such as Steedman’s, Bouin’s, and Kaiserling III. These mock fluids were used to create a comprehensive calibration data set of SORS spectral fingerprints and assess spectral changes related to chemical composition and concentration. The calibration solutions were prepared in ultrapure water (Milli-Q, Merck) with the concentrations listed in Table 1. The prepared solutions (20 mL) were measured following the protocol presented in our previous study.^18^ The concentrations represent both typical preservation fluids found in historical wet collections and potential cross-contamination scenarios caused by incorrect top-ups.

**Table 1:** Standard Fluids and Concentrations

class	type	excipients and concentrations
C1	glycerol	glycerol 5%, water 95%
C2		glycerol 35%, water 65%
C3		glycerol 65%, water 35%
C4	EtOH	EtOH 50–60%, water 50–40%
C5		EtOH 70–80%, water 30–20%
C6	EtOH and other components	EtOH 95%, MetOH 3%, water 2%
C7		EtOH 70%, MetOH 5%, water 25%
C8		EtOH 70%, MetOH 10%, water 20%
C9		EtOH 50%, formaldehyde 2%, water 48%
C10		EtOH 70%, formaldehyde 1%, water 29%
C11	formaldehyde	formaldehyde 4–5%, water 96–95%
C12		formaldehyde 4%, MetOH 1.5–2%, ∼94% water
C13		formaldehyde 1%, water 99%

class	type	excipients and concentrations	
C14	preserving fluid mock-up recipes	propylene phenoxetol	propylene phenoxetol (purity ≥93%, impurities: <7% di(propylene glycol)
C15		thymol	saturated solution in distilled water (1 g/L)
C16		Steedmans	propylene glycol 4.5%, formaldehyde 2%, propylene phenoxetol 0.5%, water 93%
C17		Kaiserling III	glycerol 25%, sodium acetate (16.7 w/v %), water 75%
C18		Bouin’s	picric acid aqueous (saturated solution ∼1.8%), formaldehyde 10%, acetic acid 5%, ∼water 83.2%
C19		propylene glycol	propylene glycol 20%, water 80%
C20		acetic acid	acetic acid 20%, water 80%

### Historic Samples from Wet Collections

The set of 46 historic specimens analyzed in this study spans a wide range and reflects taxonomic and historical diversity of the Natural History Museum’s wet collections. The specimens originated from multiple storage areas, included both vertebrates and invertebrates, such as mammals, reptiles, and fish. Historical collections included: specimens collected by Charles Darwin on the second voyage of HMS Beagle (1831-36), ex-Wellcome Trust collection specimens from the mid-20th century, and contributions from the “Tank Room” (from 19th century to present day). A subset of the samples also included experimental preservation material of unknown provenience and uncertain composition, from the early-to mid-20th century using Steedman’s, phenoxetol, or Dowicil-based formulations. The expected preservation fluids varied by taxonomic group and time period. Typical fluids used for vertebrates, particularly mammals and reptiles, include formaldehyde (4–10%) for initial fixation, followed by long-term storage in ethanol (70–80%). Invertebrate samples, especially jellyfish and shrimps, were commonly stored in formaldehyde or buffered formaldehyde, sometimes with additives like glycerol or phenoxetol to improve tissue preservation. The list of analysed samples, with expected fluid and identification, is presented in Table 2.

**Table 2:**
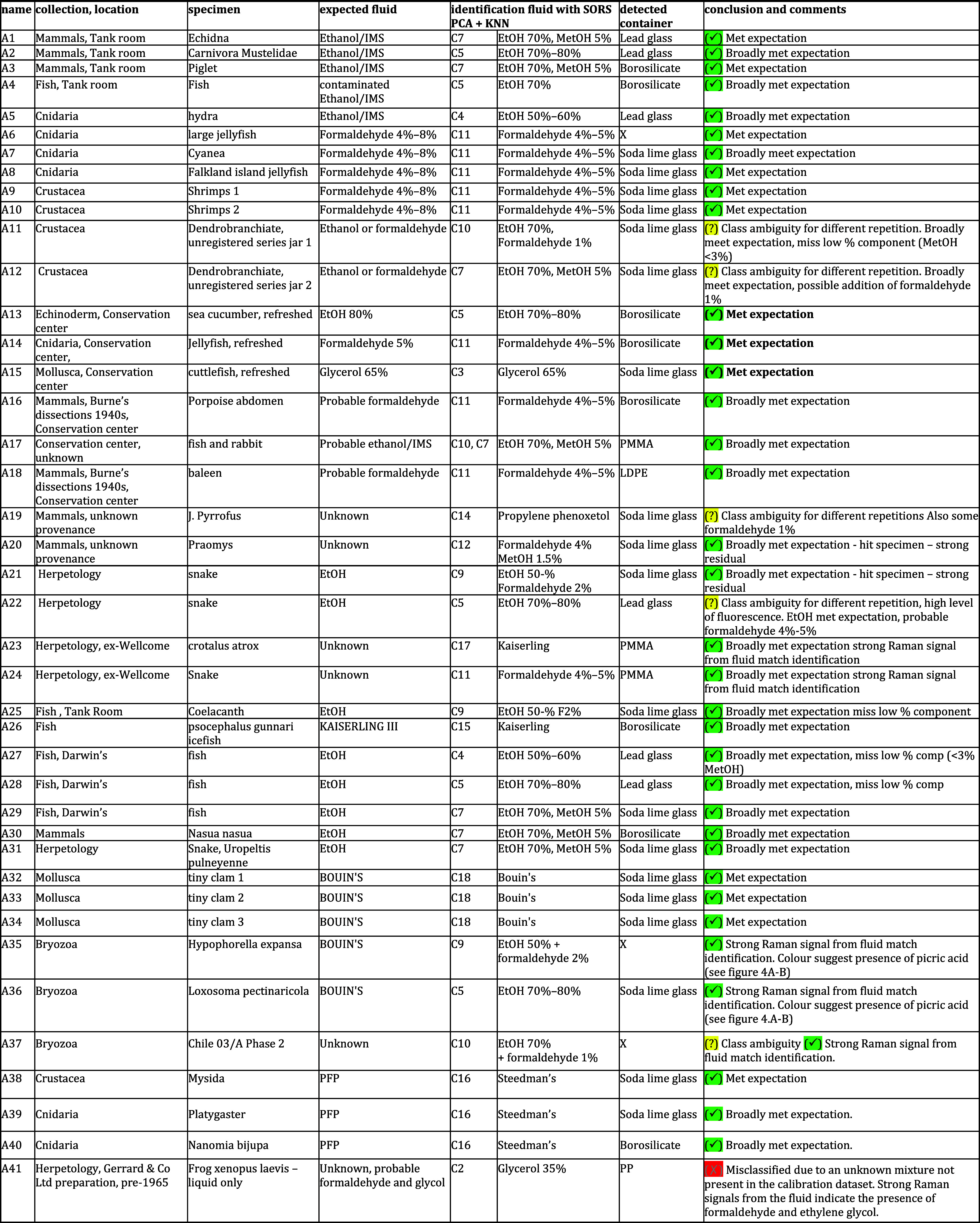

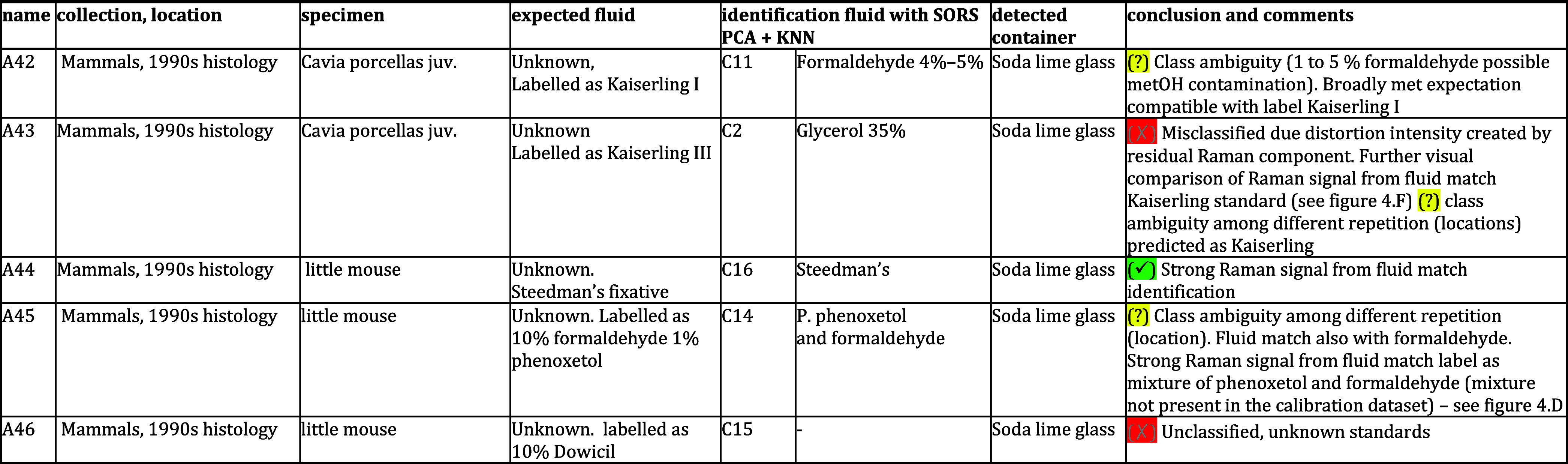
List of Historic Samples Studied from NHM Collections and Classification Results Achieved with the Proposed Method

### Instrument and Measurement Protocol

A hand-held SORS device (Resolve, Agilent Technologies, Oxfordshire, UK) was used to perform all measurements. The system employs an 830 nm laser, with a maximum output of 475 mW, operated in the “through-barrier” analysis mode. Spectral acquisition for each sample lasted 25 s in total, split between 5 s for the zero measurement (1 s × 5) and 10 s for the offset measurement (2 s × 10) collected with a 5.5 mm spatial displacement. Calibration measurements were first conducted on the mock-up solutions (C1–C20) in a controlled laboratory setting, following procedures adapted from our previous work.^18^ These were performed on separate days using a different Resolve unit and repeated on different days to assess instrument reproducibility (i.e., 6 to 24 repetitions total for each calibration class). Example of SORS spectra of the calibration fluid are shown in Figure 1A. In situ measurements (A1–A46) were then conducted directly though the historic jars and other containers at NHM, London (see Figure 1B,C). Depending on the condition and accessibility of each specimen, two to three SORS measurements were taken per specimen at different locations. All replicates for each specimen were first averaged and then analyzed, providing a more stable and representative spectrum for classification. In the second stage of analysis, the individual replicates were analyzed separately (see Supporting Information) to assess the robustness of the classification routine and to identify any potential outliers (such as those caused by accidently probing the specimen or inhomogeneity in the solution or container wall deposits).

**Figure 1:**
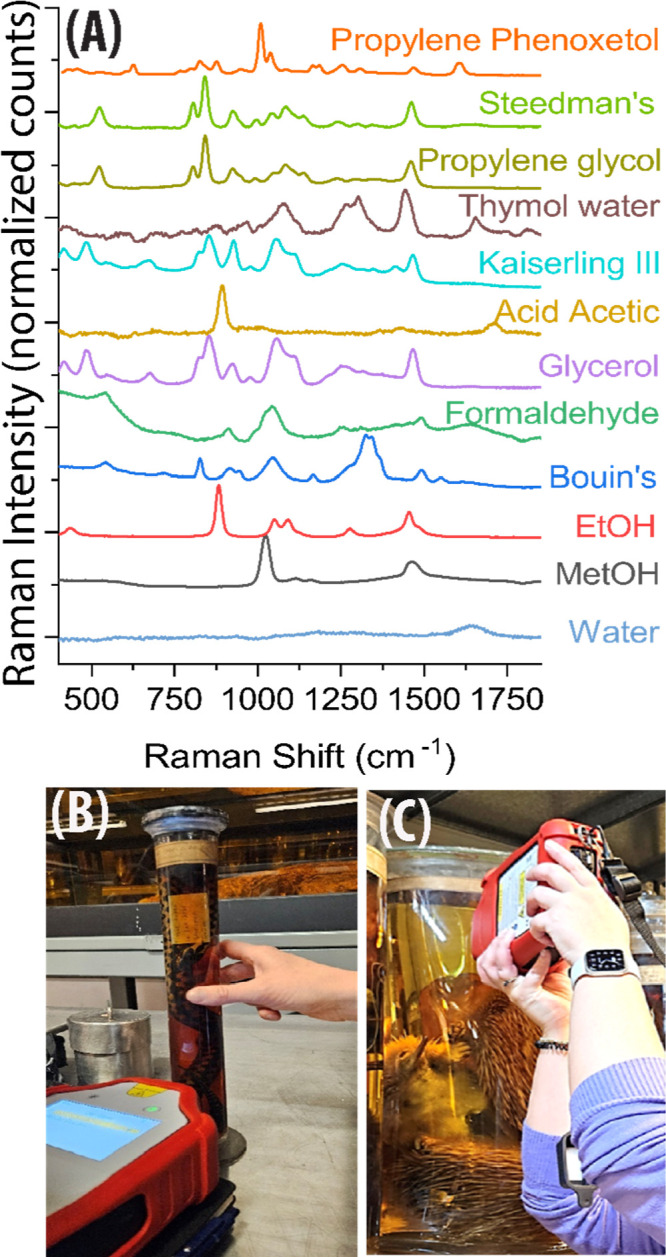
(A) Standard Raman spectra of mock-up fluid solutions used in calibrants in this study. All were measured using SORS through a glass vial. (B,C) Resolve instrument during in situ analysis showing different possible measurement configurations.

### Data Analysis

All acquired internally calibrated spectra were exported from the SORS instrument for external processing. A semiautomated analysis pipeline, developed in MATLAB, was used to process the exported data.^18^ As part of the routine workflow, the zero spectrum was rescaled using a scaling factor (SF) and subtracted from the offset measurement to minimize the fluorescence and Raman contribution from the container, isolating the chemical signature of the fluid. The resulting SORS spectra were then truncated to the 750–1800 cm^–1^ region prior to multivariate analysis (i.e., PCA, KNN-classifier, and MCR). PCA was performed on the processed spectra using Solo software (version 8.7, Eigenvector Research Inc.). Before PCA, spectra underwent baseline subtraction (5th-order polynomial fit), followed by standard normal variate (SNV) normalization. For classification of the historical samples, we applied the KNN algorithm available within Solo’s classification suite. The classifier operated in PCA space, assigning each unknown sample to the most similar class based on the Euclidean distance to its nearest neighbors (*k* = 4). Additionally, a secondary data set was generated by subtracting the offset spectrum from the zero measurement (“reverse SORS”) to isolate the spectral signature of the container material. MCR analysis was applied to the reverse-SORS data set using a non-negative matrix factorization algorithm to resolve underlying spectral components corresponding to the jar or container composition (e.g., glass, plastic). The non-negativity constraint ensures that all extracted component spectra remain physically meaningful, i.e. avoiding negative spectral features that have no physical meaning. Each component loading represents an estimated spectral profile, while the corresponding scores indicate its contribution to each measurement. This approach allows the discrimination of container materials without being influenced by signals originating from the preservation fluids.

## Results and Discussion

PCA analysis of the complete SORS calibration data set (see Table 1 for complete list and labeling) shows good discrimination between different solutions, as shown in Figure 2A–C, with the PCA eigenvectors (Figure 2A) and biplots of significant principal components presented (Figure 2B,C). The PCA eigenvectors reflect the chemical variability across the data set due to the different fingerprints of the preserving fluids. Figure 2B shows the PCA scores along two significant principal components (PC3, PC5), which allow good class separation based on their distinct chemical profiles. Each colored ellipse indicates the 95% confidence interval for each mock-up fluid class (C1–C20, as defined in Table 1).

**Figure 2:**
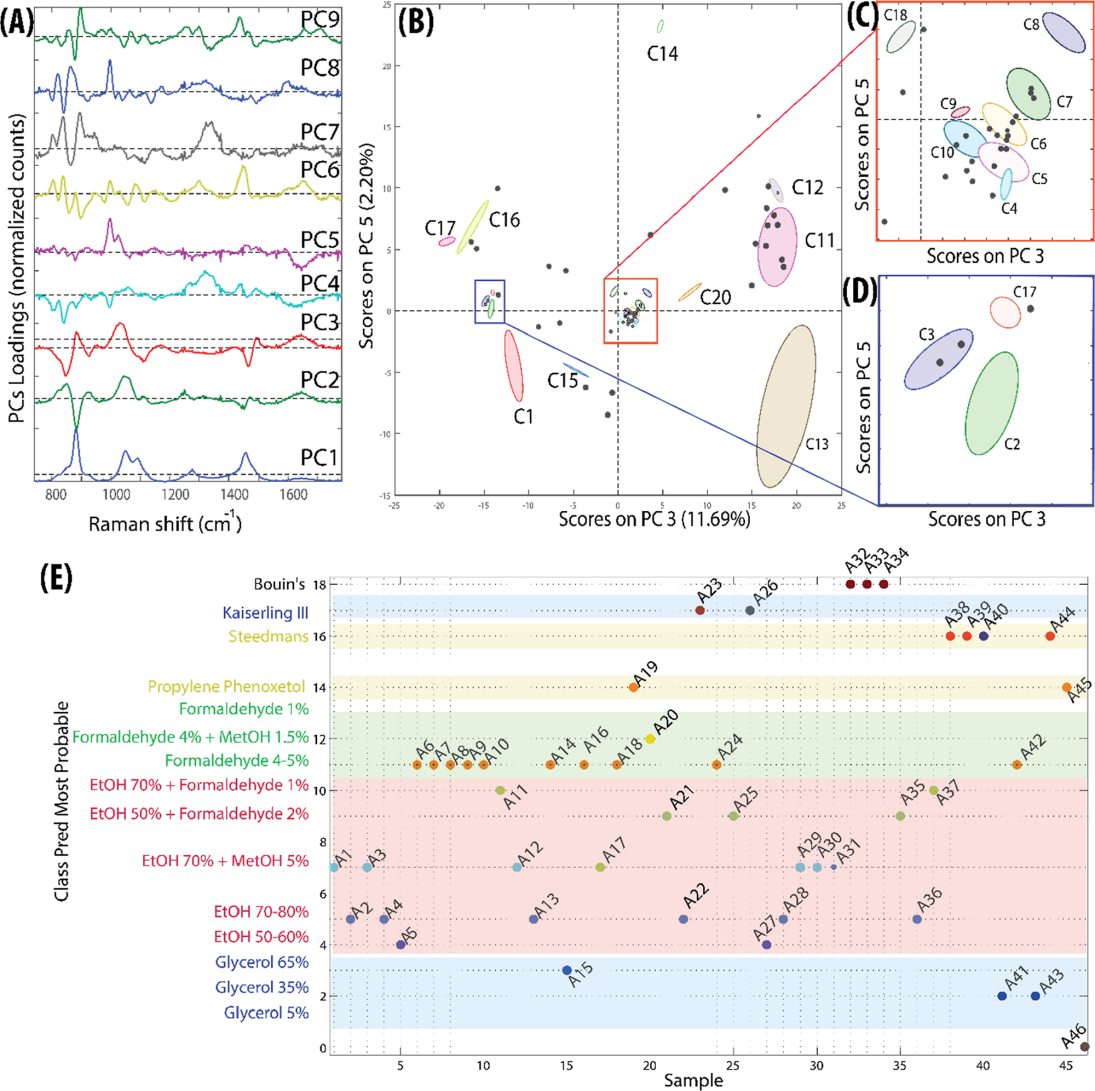
PCA and KNN-classifier results (A–C) PCA results on the mock-up solutions (C1–C20) (A) The most significant PCA eigenvectors, showing how the different Raman features contribute to a particular principal component. The *y*-axis is the normalized Raman intensity (arbitrary units). The spectra are vertically offset for clarity (normalization allows direct comparison of spectral features between samples despite variations in overall signal intensity). (B–D) PCA score plot of significant principal components showing an ability to discriminate different preservation fluids (colored ellipsoids show 95% confidence intervals for each fluid class, C1–C20). Letter labels refer to solution coding shown in Table 1. (C) EtOH–MetOH class. (D) Glycerol class. Gray dots represent the PCA scores for the historic samples (A1–A46) projected along PC3, PC5 (E) KNN-classification results for the historical samples (A1–A46). Each point represents an individual historical sample. The classification was performed using a *k*-nearest neighbors (KNN) model trained on the mock-up fluid data set (i.e., classes C1–C20). The predicted classes along the *y*-axis correspond to the most likely fluid type assigned to each historical sample.

By analyzing subsets of the mock-up fluids separately (see Supporting Information, Section SI1), we observe consistent trends in spectral features associated with different concentration levels and the presence of contaminants within the same fixative type. This targeted analysis enhances discrimination between closely related formulations. For example, in the group comprising ethanol, methanol, and various other components (C4–C10; see Figure S1.3), the samples show a clear EtOH concentration-dependent separation along PC2, with increasing ethanol content (50–60%, 70–80%, 95%) producing characteristic shifts that reflect changes in OH polarizability, as reported in our previous study.^18^ Similarly, the MetOH content is resolved along PC3, where higher concentrations (3, 5, 10%) correspond to increasing PC3 scores, indicating the distinct spectral contribution of MetOH around 1034 cm^–1^. Additionally, even low levels of formaldehyde contamination (1–2%) within 70% ethanol solutions introduce subtle but detectable Raman features, enabling discrimination along PC5. Together, these observations demonstrate that the PCA model captures the detailed chemical variability in the calibration data set, allowing robust differentiation not only between fluid types but also between different concentrations and low-level components. Historic samples (gray dots in Figure 2B–D) were projected into the same PCA model. Several of these samples clustered closely with specific preserving fluid groups. Most of the historic samples cluster near the Steedman’s (C16), formaldehyde 4–5% (C11–12), glycerol 35–65% (C2–3), Kaiserling III (C17), and ethanol–methanol mixture groups (C4–C10), suggesting a similar chemical formulation to those reference fluids. Further support comes from the KNN classification, which assigns each historic sample to its most probable fluid class based on proximity to known samples (calibration data set) in PCA space. Figure 2E presents the KNN classification results for all the historic samples (A1 to A46). These results are also summarized in Table 2, showing the predicted fixative identity for each historic sample along with sample information and expected identity. Data on the composition of the container (i.e., type of glass or plastic) was extracted using the “reverse SORS” technique described in the materials and method section to isolate the signal from the container. The resulting spectra were analyzed using MCR with non-negativity constraints. As shown in Figure 3, the MCR analysis (Figure 3A–D) reveals distinct clustering of samples according to container type. The extracted eigenvectors (Figure 3A) represent major chemical components corresponding to common glass or plastic materials; specifically, Comp 1, 2, and 3 show the typical fluorescence emission from borosilicate glass, soda lime glass and lead (Pb) glass, respectively, while Comp 4, 5, and 6 show very rich Raman spectra consistent with plastic/acrylic compositions. The MCR score plots (Figure 3B–D) show the clustering historic samples for high scores of: component 1 (A3, A4, A11, A13, A15, A26, A30, A40) corresponding to borosilicate glass (blue cluster), component 2 (A7, A8, A9, A10, A12, A14, A16, A19, A20, A21, A25, A29, A31, A32, A33, A34, A35, A36, A37, A38, A39, A42, A43, A44, A45, A46) corresponding to soda-lime glass (green cluster), and component 3 (A1, A2, A5, A22, A27, A28) lead glass (red). Figure 3E illustrates the average emission spectra for each glass type, confirming the compositional differences between clusters. Figure 3F shows the average Raman spectra for containers characterized by a high score along MCR components 4, 5, and 6, respectively, identifying poly­(methyl methacrylate) (PMMA: A17, A23, A24), low-density polyethylene (LDPE: A18), and polypropylene (PP: A41).

**Figure 3:**
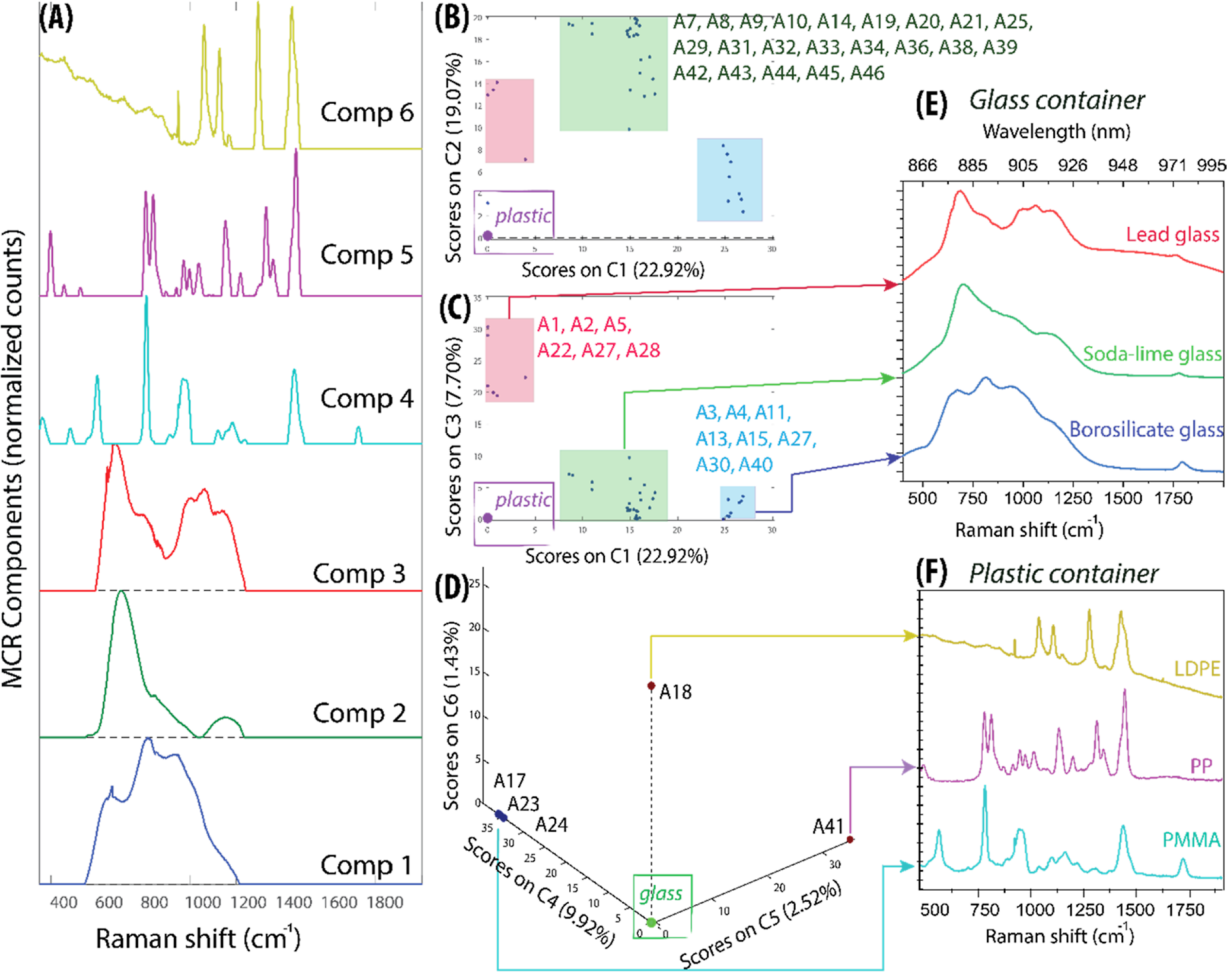
MCR results (A–D) and enhanced SORS spectra (i.e., “reverse SORS”) of containers (E,F). (A) The most significant MCR eigenvectors, showing main composition of measured historical jars. (B,C) MCR score plots of the first three components showing clear discrimination between different types of glass containers based on their spectral emission. Specifically, Comp 1 = borosilicate glass (blue), Comp 2 = soda-lime glass (green), and Comp 3 = lead glass (red). Letter labels (A1–A46) correspond to historical samples listed in Table 2. (D) 3D MCR score biplot along components 4–6, highlighting the separation of plastic containers according to their distinct spectral fingerprints. Each cluster corresponds to a different polymer type: Comp 4 = PMMA (cyan), Comp 5 = LDPE (violet), and Comp 6 = PP (orange). (E) Average emission spectra from each cluster (colored rectangle) recognized in the MCR score plot, corresponding to samples with high values of: Comp1 = borosilicate glass, blue line (blue rectangle in B,C), Comp2 = soda-lime glass, green line (green rectangle in B,C), Comp3 = lead glass, red line (red rectangle in B,C) (F) Raman spectra of plastic/acrylic containers highlighted by high score along: Comp 4 = PMMA (cyan line), Comp 5 = LDPE (violet line), and Comp 6 = PP (yellow line).

Table 2 summarizes the identification results for 46 historic specimens from the NHM collections. The method successfully identified the preservation fluid in the majority of cases. Specifically, 36 samples (78.5%) either fully met or broadly aligned with expectations based on curatorial records and visual comparisons between the sample SORS spectra and their assigned reference class (see Figure 4A). An additional 7 samples (15%) showed partial agreement but exhibited classification ambiguities across different spectral repetitions (see Figure S1.2). In some of these cases, the high fluorescence background affected relative intensity patterns, leading to slight variations in the predicted concentrations within the same fluid class. In other cases, sequential visual inspection of SORS spectra revealed the presence of low-concentration secondary components not captured by the primary classification.

**Figure 4:**
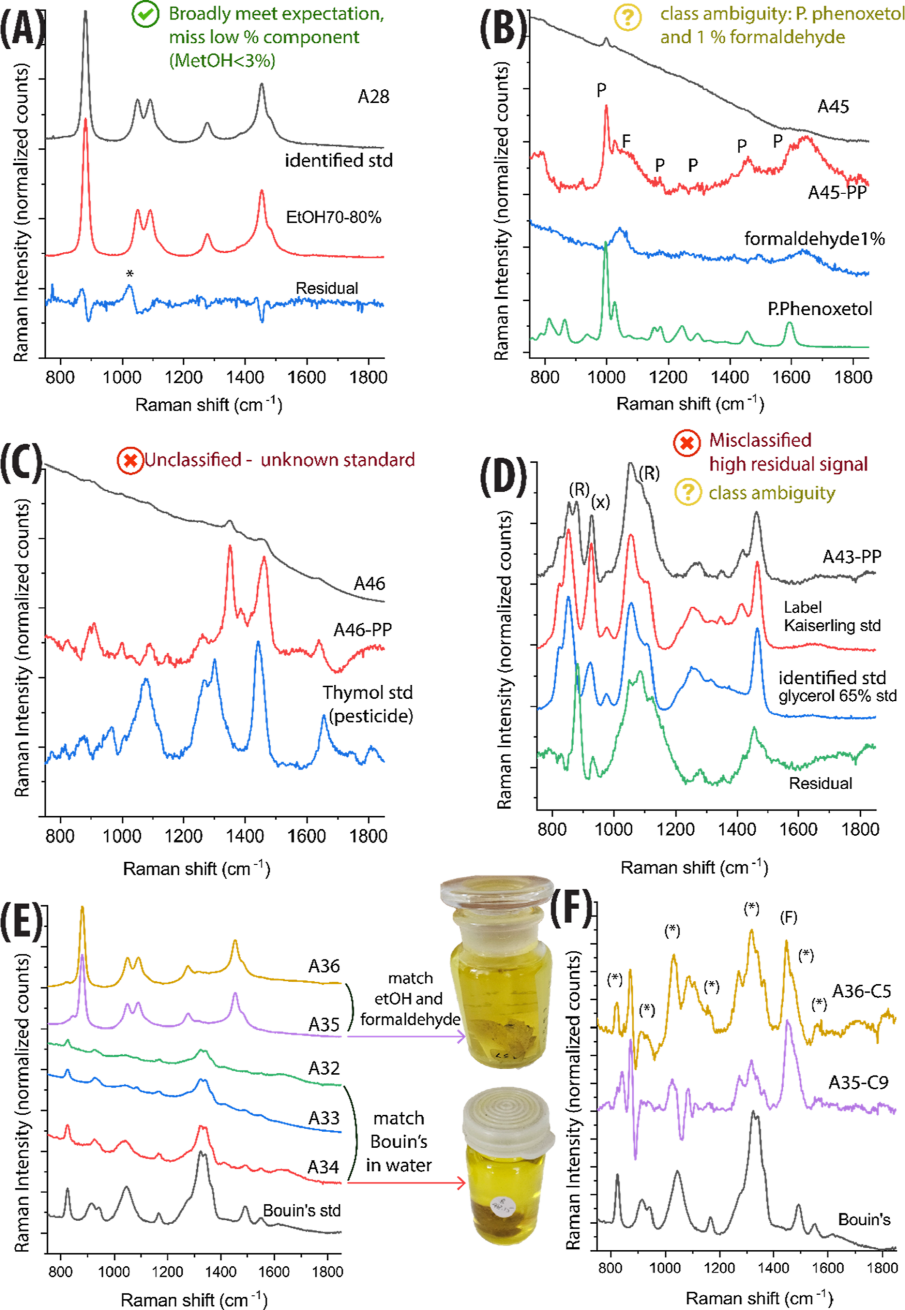
SORS spectra and classification results for selected historical specimens, illustrating correct classifications, ambiguous cases, and misclassifications. Example of: (A) (√) broadly matching expectations, missing only a low-level component (<3% MeOH). (B) (?) classification ambiguity across different measurement locations. (C) (×) Unclassified case due to the presence of an unknown fluid (not included in the reference data set). (D) (×) Misclassification caused by spectral distortion from residual Raman contributions originating from the specimen itself. “R” indicates major interfering Raman signals; “X” marks the band responsible for misclassification. (E,F) Examples of Bouin’s solutions in water and ethanol, visually indistinguishable but spectrally distinct. (F) Residual spectrum obtained by subtracting the assigned reference standard from the historic sample, with (*) highlighting residual Raman bands attributed to picric acid.

For example, Figure 4B shows the processed spectra of sample A45, with the presence of both Phenoxetol and formaldehyde. As this mixture was not included in the calibration data set, the classification based on the average spectrum reflected only the dominant signal (Phenoxetol). Nevertheless, the main solvent component was consistently identified. Only 3 cases (6.5%) were misclassified or unclassified. Sample A46, labeled as Dowicil (see Figure 4C), remained unclassified because the fixative was not included in the calibration data set. Sample A41, was misclassified as 35% glycerol. A further visual comparison with reference SORS spectra (see Figure S1.3) revealed the presence of both formaldehyde and ethylene glycol (EG), a mixture not represented in our calibration data set. Consequently, the method assigned the sample to the class with the most similar spectral features (i.e., glycerol), due to broad overlapping bands in the 1200–1600 cm^–1^ region. This similarity arises from their related chemical structures, as ethylene glycol (a diol) and glycerol (a triol) share some vibrational modes. Both these misclassifications could likely be resolved by expanding the calibration data set to include additional solutions and mixtures, thereby improving the model’s ability to capture such variability. Therefore, future work should explicitly account for these factors through expanded calibration data sets, including additional classes of materials tailored to mitigate possible ambiguous classification (e.g., pesticides, additional glycol-based substances, and a broader range of concentrations).The last case, sample A43, labeled Kaiserling III, was misclassified as a 35% glycerol solution, due to spectral distortion from residual Raman signals originating from the specimen itself. However, the visual comparison with reference SORS spectra (Figure 4.D) suggests that the sample is indeed more similar to Kaiserling III, based on the presence of an additional band at ∼ 926 cm^–1^ associated with potassium acetate. While A43 is considered misclassified, the model correctly identified the main excipient of the formulation, glycerol (35%), highlighting its ability to capture key compositional features despite limitations in spectral quality.

An illustrative example of the added value of using SORS in identifying historical fixative recipes is provided by samples A32 to A36, which all exhibit a similar yellow coloration (Figure 4E–F), typically associated with Bouin’s fluid (due to the presence of picric acid), from the same historical period and specimen type.^24^ However, SORS-based classification offered deeper insight: A32, A33, and A34 were identified as Bouin’s in water, while A35 and A36 matched ethanol-based solutions, specifically ethanol with low concentrations of formaldehyde. Despite their visual similarity, the SORS spectra of A35 and A36 show a dominant EtOH signal alongside residual picric acid features (asterisk in Figure 4F), suggesting they were likely prepared as Bouin’s in alcohol (70–80% EtOH).^4^ These results demonstrate how SORS enables the reliable differentiation of closely related or visually indistinct preserving fluids, enhancing the accuracy of historical classification beyond visual inspection alone. These findings can also offer insights into curatorial and conservation practices. The detection of compositional variants, such as Bouin’s using ethanol rather than water, suggests institutional adaptation of recipes that reflect available materials or preservation standards that have changed over time. Similarly, the presence of mixed or modified formulations (e.g., Phenoxetol with formaldehyde) may indicate undocumented maintenance or top-up interventions. Such results demonstrate how spectral analysis of preservation fluids not only aids chemical classification but also provides a valuable window into the material history and evolving curatorial strategies within museum collections.

## Conclusion

This study presents the first in situ demonstration of combining SORS with multivariate analysis for the classification of preservation fluids in historical biological collections. This study successfully demonstrates that SORS can noninvasively classify preservation fluids in sealed historical specimens, validates the role of multivariate analyses in resolving chemically complex or visually similar mixtures, and illustrates how these insights can directly support curatorial decision-making and long-term conservation strategies. It was performed at the Natural History Museum, London by analyzing 46 chemically diverse specimens spanning various collections and fixative recipes. The method was tested under realistic curatorial conditions without opening the container or preparing the sample in any way. This noninvasive and portable approach reliably identified both preservation fluid and container type, including cases where fluids appeared visually indistinguishable. It also demonstrated high sensitivity to low-concentration components and accurately recognized the main excipients even in unknown mixtures (i.e., not present in the calibration data set). Additionally, the use of "reverse" SORS combined with MCR enabled clear discrimination between different container materials, such as glass and plastics, providing insight into storage conditions, handling considerations (e.g., lead glass), and possible fluid–container interactions (e.g., type of plastic). These findings underscore the broader potential of SORS-based techniques not only for retrospective fluid classification but also for monitoring chemical changes over time. Overall, this work establishes a practical tool that enhances conservation decision-making and advances our understanding of specimen storage history across both taxonomic and temporal diversity.

## Supplementary Material



## Data Availability

Data and MATLAB script are openly available in STFC public repository eDATA^25^ available at https://edata.stfc.ac.uk/handle/edata/984.

## Notes

Discrimination of different concentration and contaminant in mock-up fluids for each type of recipe: (Figure S1.1) Glycerol based (Figure S1.2) Formaldehyde (figure S1.3) EtOH MetOH mixture. (Figure S2) KNN prediction on all different repetition within samples. SORS spectra of sample A41 (Figure S3) (PDF)

## References

[1] Domański J., Janczura A., Wanat M., Wiglusz K., Grajzer M., Simmons J. E., Domagała Z., Szepietowski J. C. (2023). Preservation
Fluids of Heritage Anatomical Specimens —a Challenge for Modern
Science. Studies of the Origin, Composition and Microbiological Contamination
of Old Museum Collections. J. Anat..

[2] Simmons, J. E. Storage in Fluid Preservation. In Preventive Conservation: Collection Storage; Elkin, L. , Norris, C. A. , Eds.; Society for the Preservation of Natural History, 2019; pp 491–509.

[3] Simmons, J. E. Fluid Preservation; Rowman & Littlefield Publishers: Lanham, 2014.

[4] Ceríaco L. M. P., Marques M. P. (2025). Fluid-Preserved
Zoological Specimens in Portuguese
Natural History Collections: A Historical Overview and Implications
to Collection Management and Research. Nat.
Hist. Collect. Museomics.

[5] Birch, T. The History of the Royal Society of London for Improving of Natural Knowledge from Its First Rise, in Which the Most Considerable of Those Papers Communicated to the Society, Which Have Hitherto Not Been Published, Are Inserted as a Supplement to the Phil; The History of the Royal Society of London for Improving of Natural Knowledge from Its First Rise, in which the Most Considerable of Those Papers Communicated to the Society, which Have Hitherto Not Been Published, are Inserted as a Supplement to the Phil; A; Millar in the Strand, 1756.

[6] Schiller E.
K., Haring E., Daubl B., Gaub L., Szeiler S., Sattmann H. (2014). Ethanol Concentration
and Sample Preservation Considering
Diverse Storage Parameters: A Survey of Invertebrate Wet Collections
of the Natural History Museum Vienna. Ann. Des
Naturhistorischen Museums Wien.

[7] Trillat A. (1892). Sur Les Propriétés
Antiseptiques de La Formaldéhyde. Bull.
la Société Philomath. Paris.

[8] Cersoy S., Rouchon V., Belhadj O., Cuisin J., Herbin M. (2020). Noninvasive
Fluid Identification: Potential of Micro-Raman Spectroscopy. Collect. Forum.

[9] Buffon, G. L. L. ; Daubenton, M. Histoire naturelle, générale et particulière, avec la description du Cabinet de roi; De l’Imprimerie royale: Paris, 1756; pp 1749–1767.

[10] Baird, S. F. Directions For Collecting, Preserving And Transporting Specimens Of Natural History, Prepared For The Use Of The Smithsonian Institution; Creative Media Partners, LLC, 1857.

[11] Culling, C. F. A. Handbook of Histopathological and Histochemical Techniques: Including Museum Techniques; Butterworth-Heinemann, 1974.

[12] McKenzie A. T., Nnadi O., Slagell K. D., Thorn E. L., Farrell K., Crary J. F. (2024). Fluid Preservation
in Brain Banking: A Review. Free Neuropathol.

[13] Burroughs G. E., Makos K., Hawks C., Ryan T. J. (2006). Exposure of Museum
Staff to Formaldehyde during Some Wet Specimen Activities. Collect. Forum.

[14] Von
Endt D. W. (1994). Spirit Collections: A Preliminary Analysis of Some
Organic Materials Found in the Storage Fluids of Mammals. Collect. Forum.

[15] Moore S. J. (1994). What Fluid
Is This?. Biol. Curators Gr. Newsl..

[16] Long, D. A. . In The Raman Effect; Long, D. A. , Ed.; John Wiley & Sons, Ltd: Chichester, UK, 2002.

[17] Lux A., Realini M., Botteon A., Maiwald M., Müller A., Sumpf B., Miliani C., Matousek P., Strobbia P., Conti C. (2024). Advanced Portable Micro-SORS Prototype Coupled with SERDS for Heritage
Science. Analyst.

[18] Mosca S., Montgomery W., McKibbin C., Stokes R., Conti C., Matousek P. (2025). Noninvasive Characterization of Preservation Fluids
through Glass Container Using Spatially Offset Raman Spectroscopy:
Potential in Heritage Science. ACS Omega.

[19] Matousek P., Clark I. P., Draper E. R. C., Morris M. D., Goodship A. E., Everall N., Towrie M., Finney W. F., Parker A. W. (2005). Subsurface
Probing in Diffusely Scattering Media Using Spatially Offset Raman
Spectroscopy. Appl. Spectrosc..

[20] Mosca S., Conti C., Stone N., Matousek P. (2021). Spatially Offset Raman
Spectroscopy. Nat. Rev. Methods Prim..

[21] Mosca S., Lin Q., Stokes R., Bharucha T., Gangadharan B., Clarke R., Fernandez L. G., Deats M., Walsby-Tickle J., Arman B. Y., Chunekar S. R., Patil K. D., Gairola S., Van Assche K., Dunachie S., Merchant H. A., Kuwana R., Maes A., McCullagh J., Caillet C., Zitzmann N., Newton P. N., Matousek P. (2023). Innovative Method for Rapid Detection
of Falsified COVID-19 Vaccines through Unopened Vials Using Handheld
Spatially Offset Raman Spectroscopy (SORS). Vaccine.

[22] Shehata M., Dodd S., Mosca S., Matousek P., Parmar B., Kevei Z., Anastasiadi M. (2024). Application of Spatial Offset Raman
Spectroscopy (SORS) and Machine Learning for Sugar Syrup Adulteration
Detection in UK Honey. Foods.

[23] Mosca, S. ; Matousek, P. SORS spectra of preservation fluids through different glass-type containers; eData, STFC, 2025.

[24] Carter, J. ; Simmons, J. ; Crimmen, O. ; Neumann, D. Best Practices in the Preservation and Management of Fluid-Preserved Biological Collections; Society for the Preservation of Natural History, 2022.

[25] Mosca, S. ; Matousek, P. In Situ SORS Spectra of Historical Preservation Fluids in Sealed Containers; STFC eData, CLF Datasets, 2025.

