# Supplementary material for: In Situ Analysis of Historical Preservation Fluids in Sealed Containers with Spatially Offset Raman Spectroscopy
Source: ACS Omega. 2026 Jan 13;11(3):4216–25. doi: 10.1021/acsomega.5c09045 (PMC12854593; doi:10.1021/acsomega.5c09045)
Supplement: Supplementary file 1 [file ao5c09045_si_001.pdf]

Supplementary Information

In Situ Analysis of Historical Preservation Fluids in Sealed Containers with Spatially Offset Raman Spectroscopy

Ana Blanco[1], Wren Montgomery*[2], Sam Walker[1], Chelsea McKibbin[2], Robert Stokes[1], Pavel Matousek[3], Sara Mosca*[3,4]

1 Agilent Technologies LDA UK, Becquerel Avenue, Didcot OX11 0RA, UK

2 Science Innovation Platforms, Department of Science, Natural History Museum, Cromwell Road, London SW7 5BD, UK

3 Central Laser Facility, Research Complex at Harwell, STFC Rutherford Appleton Laboratory, UKRI, Harwell Campus, OX11 0QX, UK

d Department of Physics and Astronomy, University of Exeter, Exeter EX4 4QL, United Kingdom

Corresponding Author:

* Wren Montgomery, NHM: wren.montgomery@nhm.ac.uk

* Sara Mosca, STFC: sara.mosca@stfc.ac.uk

Table of contents:

S1: Discriminating between different concentrations and contaminants in mock-up fluids for each type of recipe

S1.1 Glycerol-based page 3

S1.2 Formaldehyde page 4

S1.3 EtOH-MeOH mixture page 5

S2: KNN predictions of all different repetition within samples. page 6-7

S3: SORS Spectra of sample A41 page 8

S1: **Discrimination of different concentrations and components in calibration fluids for each recipe type**

S1.1 Glycerol-based: C1, C2, C3, C17

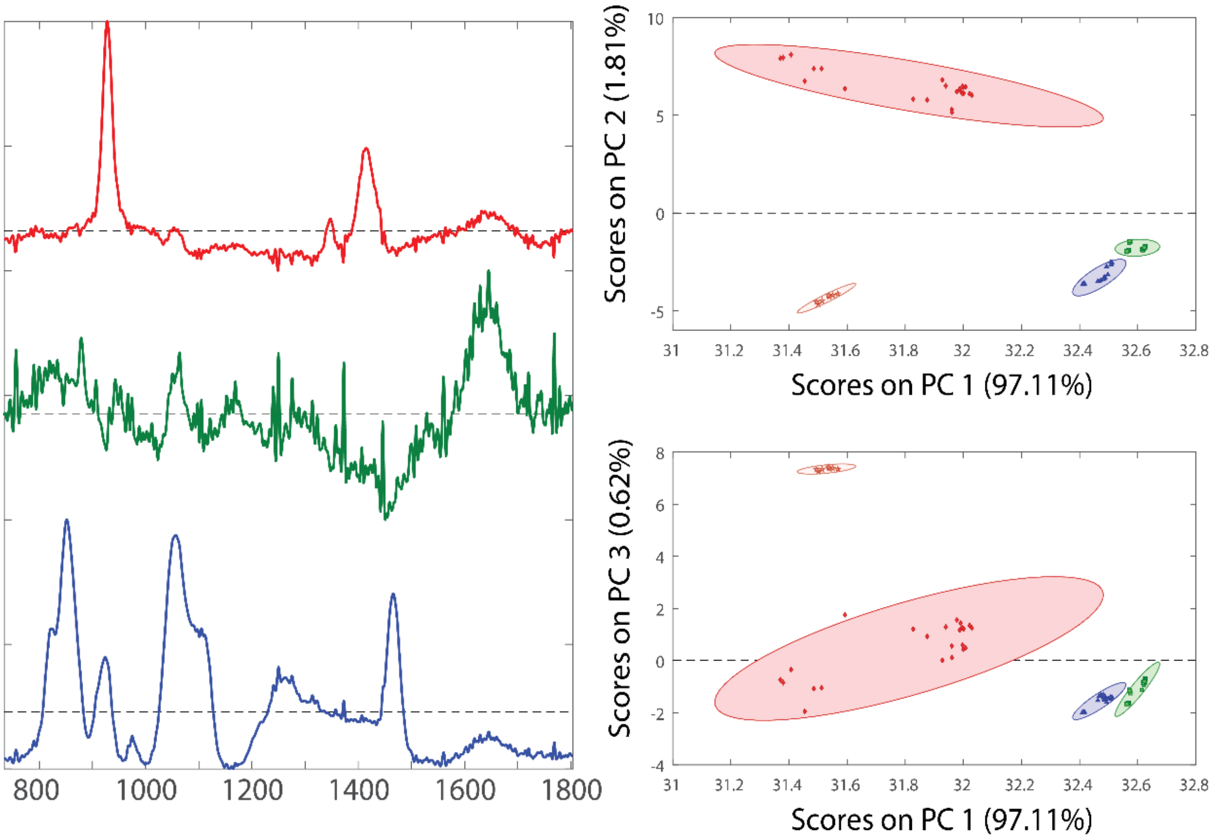

Figure S1.1: PCA results for sub-datasets containing different concentrations of glycerol in water and Kaiserling III.

The PCA score plots (right panel) show clear separation along PC1 and PC2 based on glycerol concentration, enabling discrimination between solutions ranging from 35% to 65% glycerol. PC3 enables further separation of Kaiserling III samples due to the presence of sodium acetate. The corresponding PCA loading vectors (left panel) highlight the spectral regions contributing most to this variability, indicating the sensitivity of the method to subtle compositional differences.

S1.2 Formaldehyde: C11, C12, C13

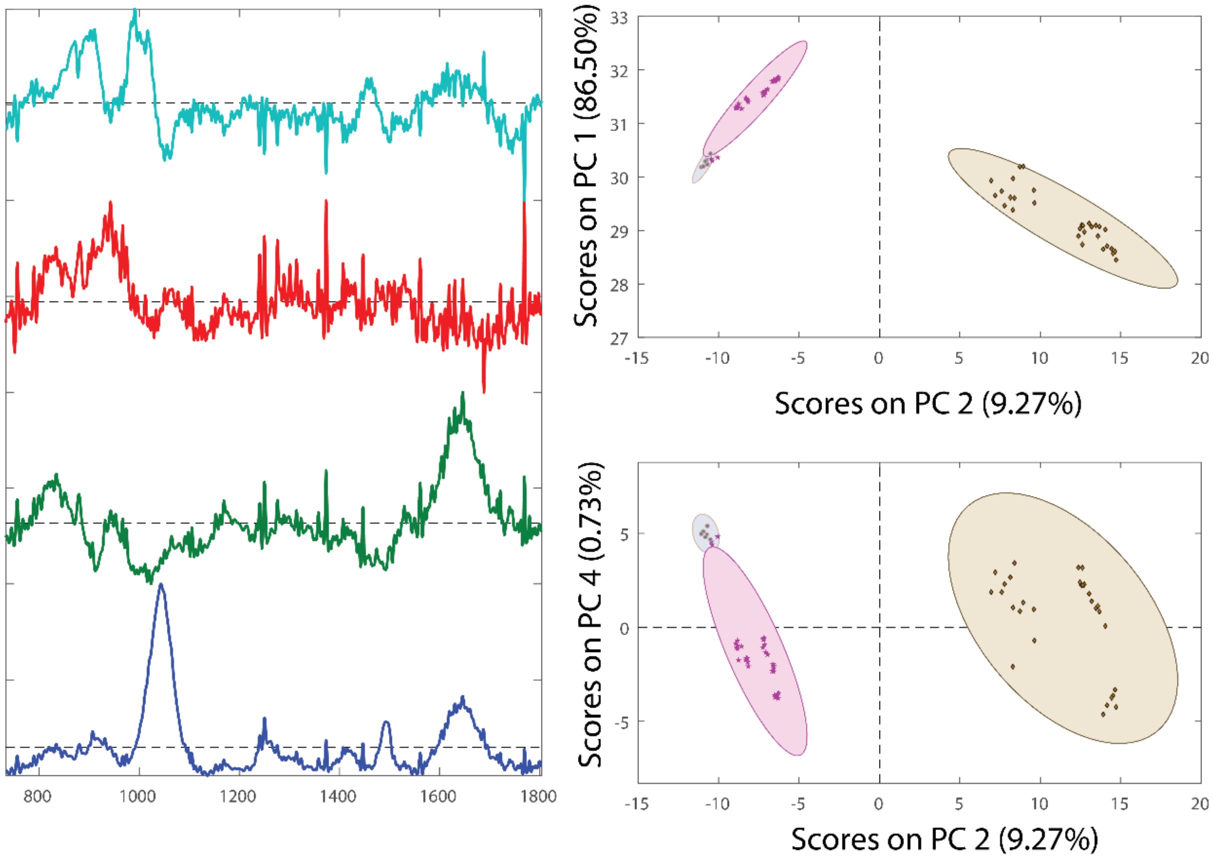

Figure S1.2: PCA results for formaldehyde-based fluids, including aqueous formaldehyde and formalin (formaldehyde 4% + methanol 1.5%).

The **PC1 vs. PC2** score plot (right) enables discrimination based on formaldehyde concentration and water content, differentiating aqueous solutions from formalin. Separation along **PC4** further distinguishes between formalin with and without methanol, primarily due to a shoulder at **1034 cm^{-1}** associated with methanol content.

S1.3 EtOH, MetOH and contaminants: C4-C10

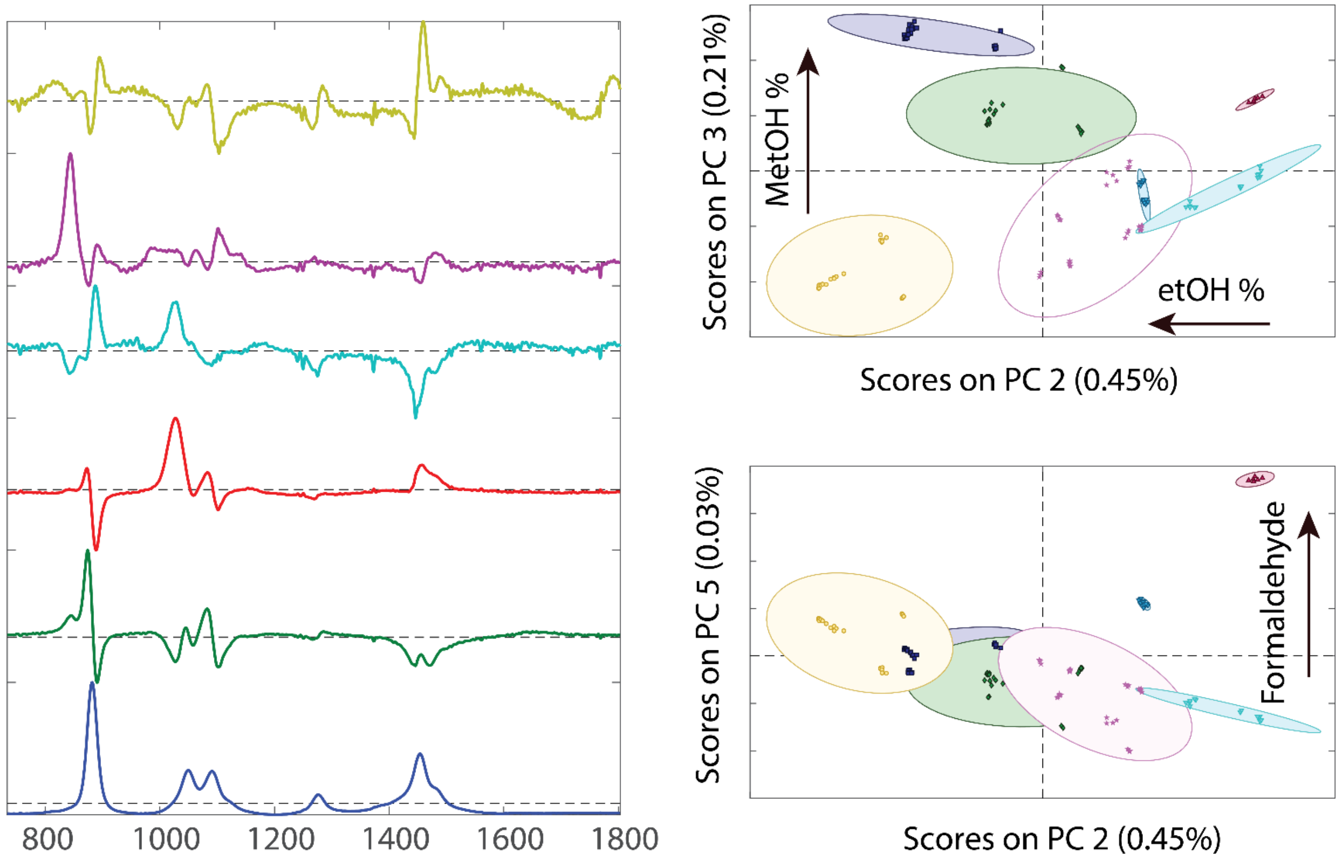

Figure S1.3: PCA results for ethanol-based mixtures containing methanol and formaldehyde.

The **eigenvectors** (left) show that the discrimination between samples at different concentrations is based on chemical information contained in the spectra. The PCA score plots are shown in the right panel. The **PC2 vs. PC3** score plot (top right) illustrates the concentration-dependent separation of ethanol (along PC2) and methanol (along PC3). The **PC2 vs. PC5** plot (bottom right) highlights the discrimination between **1% and 2% formaldehyde** in a 70% ethanol matrix.

S2: **KNN-classification: fluid prediction on all individual repetitions within samples**

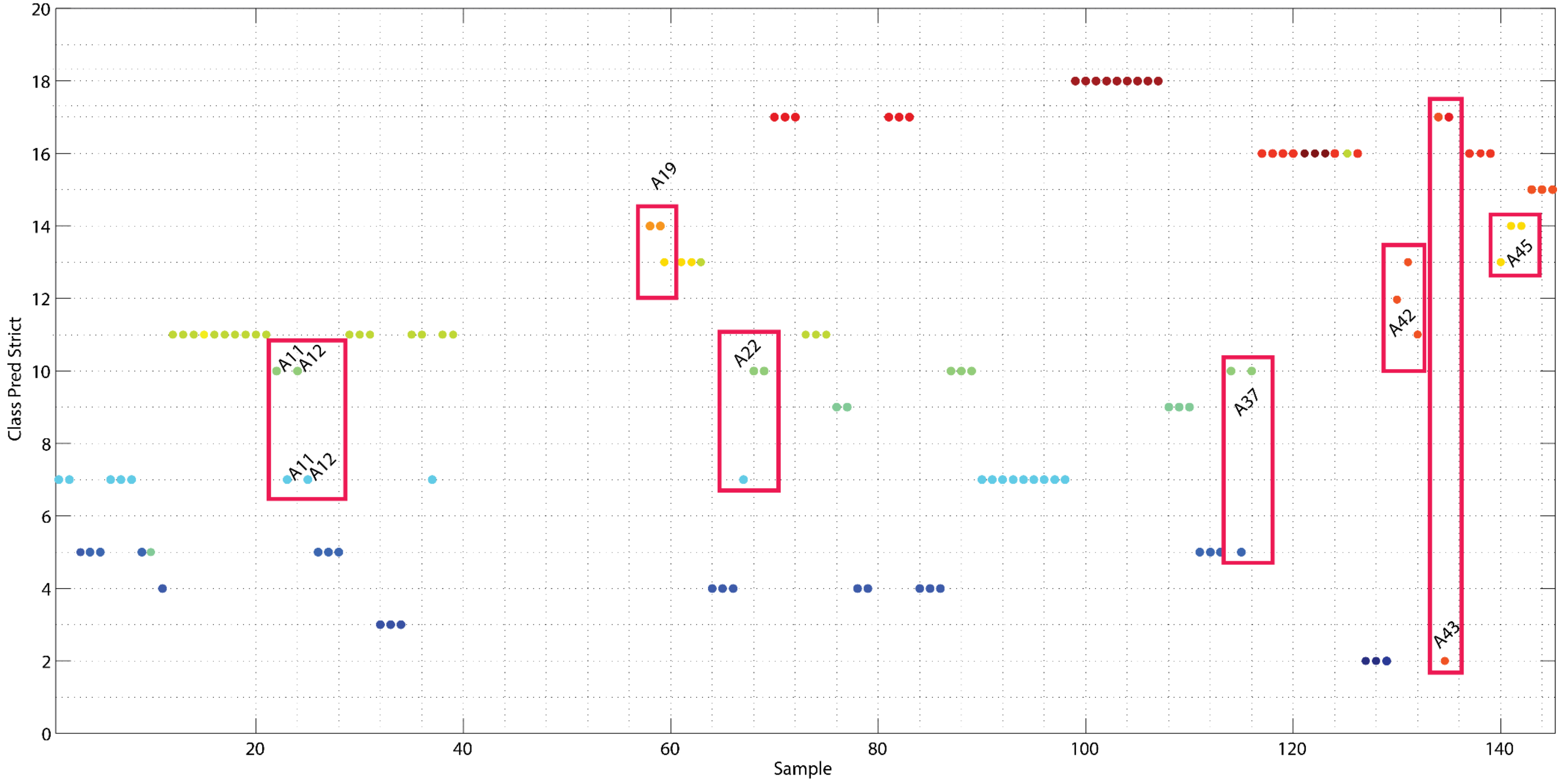

Figure S2: KNN classification results for all spectral repetitions across the SORS dataset.

Each point represents an individual prediction based on each SORS acquisition from a historic sample. The most probable fluid class (see Table 1 for class references) is shown on the y-axis. Historic samples are organized along the x-axis, grouped by specimen ID (see Table 2 for details). For most samples, predictions are consistent across repetitions. However, **red rectangles** highlight **cases of classification ambiguity**, where different repetitions produced variable class predictions. These discrepancies are discussed in the main text; typically, they arise from factors such as spectral noise, background fluorescence, or sample heterogeneity. Overall, this figure demonstrates the robustness of the KNN model while identifying a small subset of samples requiring further visual or residual spectral analysis or justify more invasive analysis techniques.

S3: SORS Spectra of sample A41 illustrating misclassification due to absence of reference in the calibration dataset

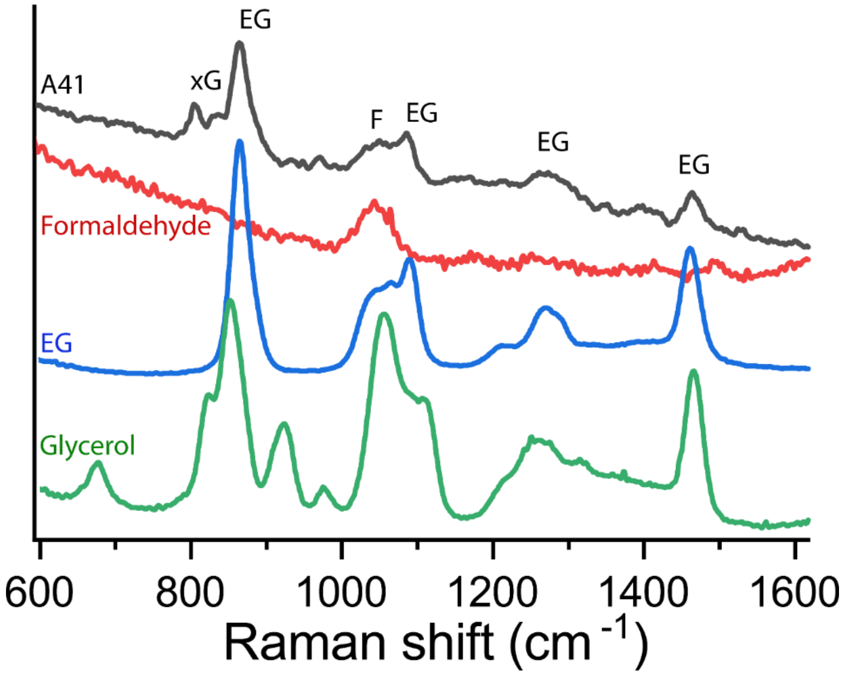

Figure S3. SORS spectra of sample A41(Black line) illustrating misclassification as 35% glycerol due to the absence of an appropriate reference in the calibration dataset. The sample spectrum is shown alongside reference spectra of formaldehyde (red line), ethylene glycol (EG – blue line), and glycerol (green line), highlighting the spectral similarities that led to the misclassification.
